# Multiscale Entropy Analysis of Page Views: A Case Study of Wikipedia

**DOI:** 10.3390/e21030229

**Published:** 2019-02-27

**Authors:** Chao Xu, Chen Xu, Wenjing Tian, Anqing Hu, Rui Jiang

**Affiliations:** 1 School of Mathematics and Computer Science, Wuhan Textile University, Wuhan 430200, China; 2 Accounting College, Wuhan Textile University, Wuhan 430200, China; 3 Department of Electrical Engineering and Computer Science, University of Michigan, Ann Arbor, MI 48109-2122, USA

**Keywords:** human behavior, complexity, page view, multiscale entropy, sample entropy, Wikipedia

## Abstract

In this study, the Wikipedia page views for four selected topics, namely, education, the economy/finance, medicine, and nature/environment from 2016–2018 are collected and the sample entropies of the three years’ page views are estimated and investigated using a short-time series multiscale entropy (sMSE) algorithm for a comprehensible understanding of the complexity of human website searching activities. The sample entropies of the selected topics are found to exhibit different temporal variations. In the past three years, the temporal characteristics of the sample entropies are vividly revealed, and the sample entropies of the selected topics follow the same tendencies and can be quantitatively ranked. By taking the 95% confidence interval into account, the temporal variations of sample entropies are further validated by statistical analysis (non-parametric), including the Wilcoxon signed-rank test and the Mann-Whitney *U*-test. The results suggest that the sample entropies estimated by the sMSE algorithm are feasible for analyzing the temporal variations of complexity for certain topics, whereas the regular variations of estimated sample entropies of different selected topics can’t simply be accepted as is. Potential explanations and paths in forthcoming studies are also described and discussed.

## 1. Introduction

We are entering an era of big data, in which the datasets we work with are characterized by the 4 Vs: volume, velocity, variety, and veracity (where veracity emphasizes the uncertainties of data) [1,2]. The statistics underlying data are consequently crucial to making the data valuable and results worthwhile, notably for large volumes of data. Traditional statistical methods, like 1st and 2nd moment statistics (the mean value and variance, respectively) or the probability density function (PDF), often ignore data’s temporal and spatial characteristics, and are even invalid in some special cases. For instance, complex physiologic time series having the same mean value or variance often contain different information [3,4]. Once these methods are applied, nuance can be lost and misunderstandings may occur. For medical applications, such misunderstandings can be fatal [4].

The multiscale entropy (MSE) algorithm [5,6] was first introduced to analyze the complexity of biological time series, in which an original time series is coarsely divided into many subseries and the sample entropy [7,8] of each subseries is calculated separately. Compared to traditional statistical methods, the MSE algorithm exhibits several advances and strengths [9,10,11]: (1) It emphasizes the temporal correlation of series elements; (2) Multiscale processing ensures that the data is deeply mined; (3) In signal processing, the averaging process that occurs as part of coarse-grained integration of the subseries can be regarded as a low pass filter and effectively eliminates noise or interference.

Website page views, one well-known set of big data, are widely touted for their potential to reflect public interest in a subject [12,13]. However, most page view data are hidden by enterprises as commercial secrets, and are inaccessible to common users. Openly authorized page view data from organizations like Wikipedia and Google are thus frequently consulted and used for commercial purposes and data mining applications. For example, market prediction or consumption style analysis [13,14,15,16]. Daily human activities dominate page views. Thus, page view series are then endowed with a variety of temporal characteristics. Many studies have addressed the statistical properties of page views [13,14,15,16], although no research has yet highlighted the temporal characteristics of page views. Meanwhile, many widely used and well-performing techniques have been introduced to analyze the big data of web searching, for example, the clustering, SVM, etc. [17,18,19,20,21,22]. However, on this topic, the angle of entropy had never been investigated in previous studies.

Motivated by this gap in the literature, and with the aim of developing a comprehensive understanding of human behavior by taking advantages of MSE algorithms, we examine the complexity of page views in this study. Considering that website page views are commonly affected by commercial actions, as in the case of China’s Internet “water armies”(people who search website for earning money or driving by commercial activities) [23], we therefore analyze Wikipedia searches, as page views of a given Wikipedia topic are highly reliable (given the importance of veracity in big data) and these searches are dominated by human intentions, rather than robotic or automatic page views. Without loss of generality, in this study, the page views (search times) of four selected topics from the years 2016–2018 were collected and then given as the input into a short-time series multiscale entropy (sMSE) algorithm to investigate their complexity.

This paper is organized as follows. Section 2 briefly introduces the basics of the sMSE algorithm. Section 3 describes the characteristics of the Wikipedia data, as well as its acquisition and processing. Section 4 calculates and discusses the sample entropies of page views of the selected topics, including their temporal characteristics, compares sample entropies across the selected topics for each year, and validates the sample entropies by using statistical analysis. Finally, Section 5 concludes the paper and suggests paths for forthcoming studies.

## 2. sMSE Algorithm

The original MSE algorithm contains two main procedures: coarse-grained division and sample entropy calculation [5,6,7,8]. MSE variants, in which one or both steps are modified or replaced, are widely applied to meet the needs of different series or signals [24]. In particular, the sMSE algorithm [25] selected in this study is ideal for the short length of page view series. For short time series, the modifications included in the sMSE algorithm are conducted as follows.

During coarse-grained division, factor *p* is induced and is effective in eliminating potential fluctuations in sample entropy [7,8]. Coarse-grained division in the sMSE algorithm is defined as

(1) $$y_{j}{}^{\left(\tau )(p\right)}=\frac{1}{\tau }\sum_{i=(j-1)\tau +1+p}^{j\tau +p}x_{i}\;1\leq j\leq (N-p)/\tau$$

in which *y* and *x* denote the elements of the coarse-grained and original series, respectively, *τ* is a scale factor, *i* and *j* represent the element IDs of series {x} and $\left\{y^{\left(\tau )(p\right)}\right\}$, and *N* is the length of original series {x}. In addition, *p* fulfills 0≤p≤τ−1.

Equation (1) and the range of *p* show that each scale factor *τ* corresponds to *τ* coarse-grained subseries that are produced by *p*. The sample entropy from the original MSE algorithm is then redefined in the sMSE algorithm as

(2) $$SE(x,\tau ,m,r)=\frac{1}{\tau }\sum_{p=0}^{\tau -1}S_{E}(y^{\left(\tau )(p\right)},m,r)$$

in which *SE* is the sample entropy of scale factor *τ* and *S*_E_ denotes the sample entropy of the *p*^th^ coarse-grained subseries, *m* is the shortest length between points, and *r* denotes the threshold in sample entropy algorithm. The sample entropy in Equation (2) is defined as

(3) $$S_{E}(m,r)=\underset{N\to \infty }{\lim}-\ln\frac{A_{m}(r)}{B_{m}(r)}$$

and is generally estimated using

(4) $$S_{E}(m,r)=-\ln\frac{A_{m}(r)}{B_{m}(r)}$$

in which $A_{m}(r)$ stands for the probability that two sequences match for m+1 points and $B_{m}(r)$ denotes the probability that two sequences will match for *m* points, with both the tolerance *r* and self-match sequences excluded. More details on $A_{m}(r)$, $B_{m}(r)$, and the sample entropy algorithm can be found in [7,8].

In general, *r* is set to 0.15 times the standard derivation of the series. However, it is worth noting that the decreasing of sample entropy in the MSE algorithm is determined by both the selection of tolerance *r* and the coarse-graining produced coherence of elements in data series, especially the former one. In many cases [26,27], for enhancing accuracy and eliminating potential errors introduced by selecting a certain tolerance *r*, the MSE algorithm is even refined by adopting an adaptive threshold *r* as a function of the scale factor in coarse-graining process. In this study, for simplification, and for focusing on the novel background we choose, we follow the original MSE algorithm [5,6,7,8] and set m=2 and r=0.15∗std.

**Figure 1:**
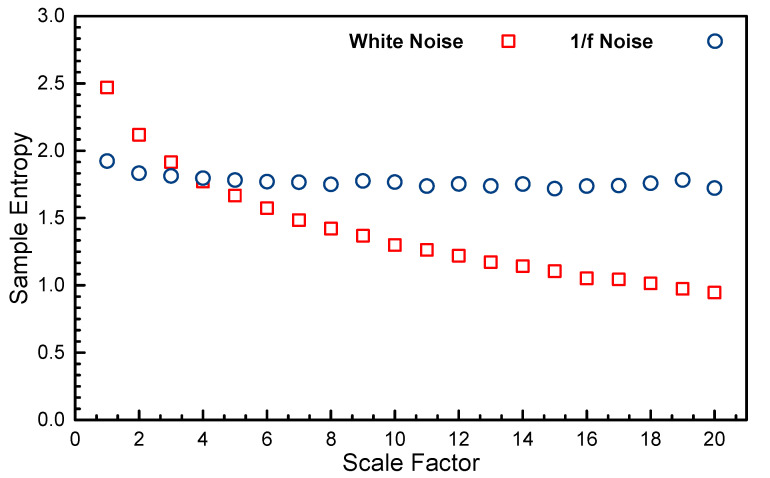
The calculated sample entropies of the white noise and 1/f noise series using the series multiscale entropy (sMSE) algorithm (series length: 1024, *m* = 2, *r* = 0.15 ∗ std).

By using the sMSE algorithm, the sample entropies of the white noise series and the 1/f noise series are calculated and shown in Figure 1. The independent elements in the white noise series tend correlate to each other, due to the averaging process in coarse-graining, in which the coherence between elements is consequently strengthened along with an increasing scale factor, and the sample entropies are therefore monotonically decreased with an increasing scale factor. On the other hand, the invariant sample entropies at all scale factors of the 1/f noise series are due to its special self-like property (fractal) [28], whose geometric shape won’t be changed at all scale factors. The results in Figure 1 agree with those of the original MSE algorithm results in [5], both quantitatively and qualitatively. The correctness and accurateness of the sMSE algorithm are therefore guaranteed and validated in this study.

## 3. Data Acquisition and Processing

**Figure 2:**
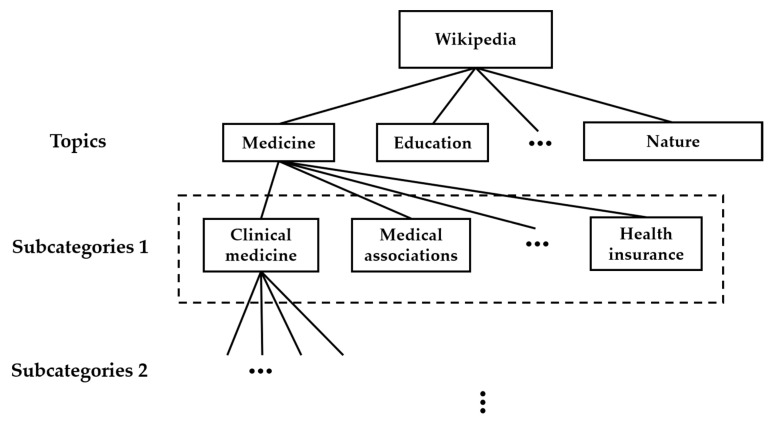
Data classification and the multilevel structure of Wikipedia.

Wikipedia content contains many topics, each divided into many subcategories, which may be further divided depending upon their intricacy. This division continues until reaching a given intricacy standard. For instance, as depicted in Figure 2, the topic of medicine is divided into many Level 1 subcategories, including clinical medicine, health insurance, and medical associations, etc. Each Level 1 category is further divided into subcategories (Level 2), until reaching given intricacy standards, and so forth. For simplification, further divisions are replaced by an ellipsis in Figure 2.

In this paper, we are only concerned with the page views of the topics named in Figure 2. We find the sum of the page views of subcategories to determine the total page views of the corresponding topic. As shown in Figure 2, the page views of the medicine topic are the sum of the page views of its Level 1 subcategories, the page views of each subcategory in Level 1 are the sum of the page views of its Level 2 subcategories, and so forth. Four topics—education, economy/finance, medicine, and nature/environment—were selected in this study for their significant impact on human lives, and their wider significance to the governments of developing countries pursuing sustainable development, for example, China [29,30,31,32,33]. Page views of the four selected topics in three recent years (2016–2018) were downloaded from https://tools.wmflabs.org/massviews/. Table 1 presents the basic data of the four selected topics, including the number of subcategories in Level 1, the length, and the mean value of the page view series of each selected topic. Because this download was conducted on 25 December 2018, the length of page view series for 2018 is therefore 359.

**Figure 3:**
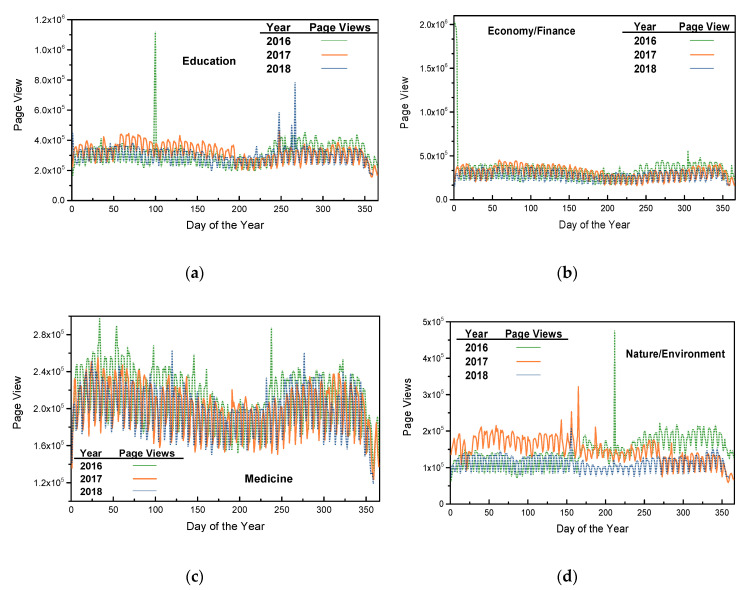
Page views of the four selected topics in 2016, 2017 and 2018: (**a**) Education; (**b**) Economy/Finance; (**c**) Medicine; (**d**) Nature/Environment.

To illustrate the temporal variations of the page view series, Figure 3 depicts the page views of the four selected topics as a function of days of the year. Figure 3 shows that, for each selected topic, the curves are close to each other, which make them indistinguishable at first glance, with only random outliers. This characteristic can be found quantitatively in Table 1, where for some topics, the mean values of page view series across the three years tended to be close. Despite these similar mean values, the three page view curves of each selected topic fluctuate rapidly, highlighting temporal fluctuations. The 1st or 2nd moment statistics and the temporal connections of page views may not be suitable for revealing connections. In addition, the nature/environment topic has the fewest page views, which reveals that environmental problems are rarely researched compared to the other three selected topics.

## 4. Results and Discussion

In biological applications of the MSE algorithm, sample entropy is physically defined as the adaptability of organisms to a certain circumstance or ecosystem [5], in which a larger sample entropy corresponds to a higher complexity and a stronger adaptability. Following this viewpoint, in this study, we define sample entropy as the complexity of human website searching activities, as well as its internal and temporal connections. That is, a larger sample entropy denotes higher complexity of human website searching activities, and elements in the page view series are less interconnected and have a weaker temporal correlation. Notice that 1st order sample entropy, or even sample entropies at small scale factors, may be invalid or insufficient to reveal hidden information conveyed by time series [3,4]. Therefore, in this section, we examine scale factors ranging from 1 to 10, as the sample entropy at scale factor 10 approaches zero. Hence, in this study, we emphasize sample entropies at large scale factors, which are also highlighted in the MSE algorithm.

**Figure 4:**
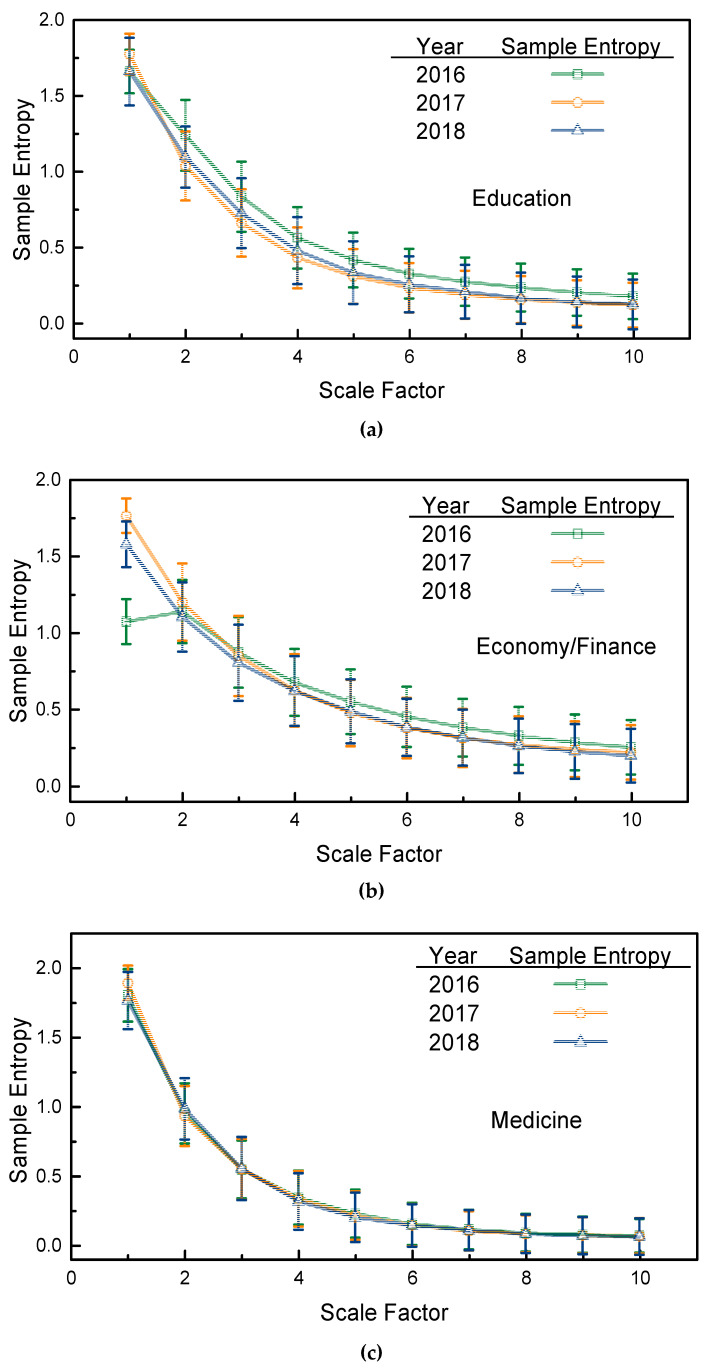

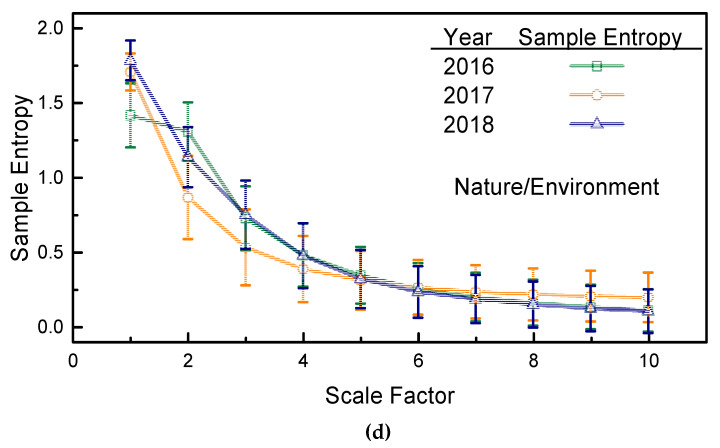
Sample entropies of page views of the four selected topics: (**a**) Education; (**b**) Economy/Finance; (**c**) Medicine; (**d**) Nature/Environment.

For each topic, the sample entropy (with a 95% confidence interval) is first calculated using the sMSE algorithm, as depicted in Figure 4. Macroscopically speaking, in Figure 4, the sample entropies of the four selected topics exhibit the same tendencies, i.e., for each selected topic, the three years’ sample entropies decrease as the scale factor increases. Compared with the sample entropies of the white noise series in Figure 1, the sample entropies of the selected topics in the three years are smaller at all scale factors and decrease more rapidly. Such a characterization fully reflects a lower complexity of the page view series of the selected topics, and the elements in the page view series are highly correlated when compared with a white noise series whose elements are independent of each other.

Specifically, for the topic of education, page views from the year 2016 appear to be the most complex, since they have the largest sample entropies at scale factors of 2 and above, whereas the page views of the year 2018 have the lowest complexity and the strongest temporal interconnections, although it has the largest 1st scale sample entropy. For the topic of economy/finance, page views from 2016 had the smallest sample entropies at small factors, but had the largest sample entropies at large scale factors (greater than 3). The page views from 2017 and 2018 had the same sample entropies at scale factors greater than 3, which somehow shows that these two years exhibited the same complexity and temporal correlations of human website searching activities. An interesting phenomenon was found for the topic of medicine: for all the years examined, the sample entropies tended to be the same at all scale factors, which reflects that in three recent years, the page view series of medicine topics have the same complexity and temporal correlations. It is not clear whether this is because the topic of medicine widely concerns all people, which would make the variations in recent years highly regularized. Lastly, for the topic of nature/environment, the sample entropies of 2016 and 2017 fluctuate as the scale factor increases, and the page views of the year 2017 had the largest sample entropies at scale factors greater than 5, whereas the page views of 2016 and 2018 had the same sample entropies at scale factors of 3 and above.

Although some selected topics, such as nature/the environment, tended to have irregular sample entropies, this could not be easily concluded when compared with other topics. However, the results in Figure 4 show that, for irregularly varied page view series, the sMSE algorithm affords an alternative analysis method and reveals the complexities and temporal correlations of page views in different years.

**Figure 5:**
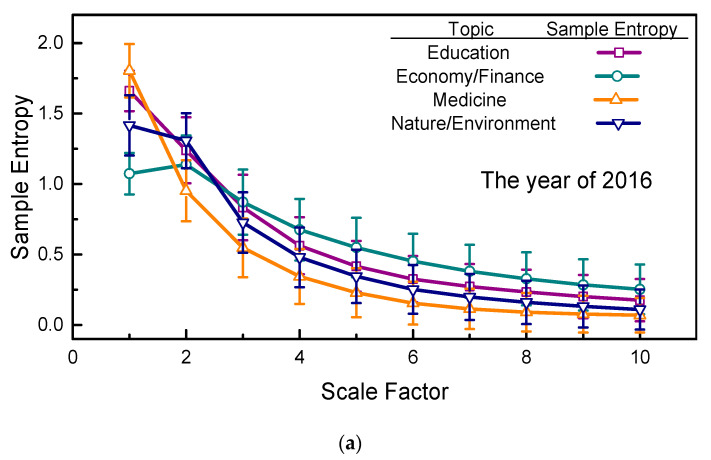

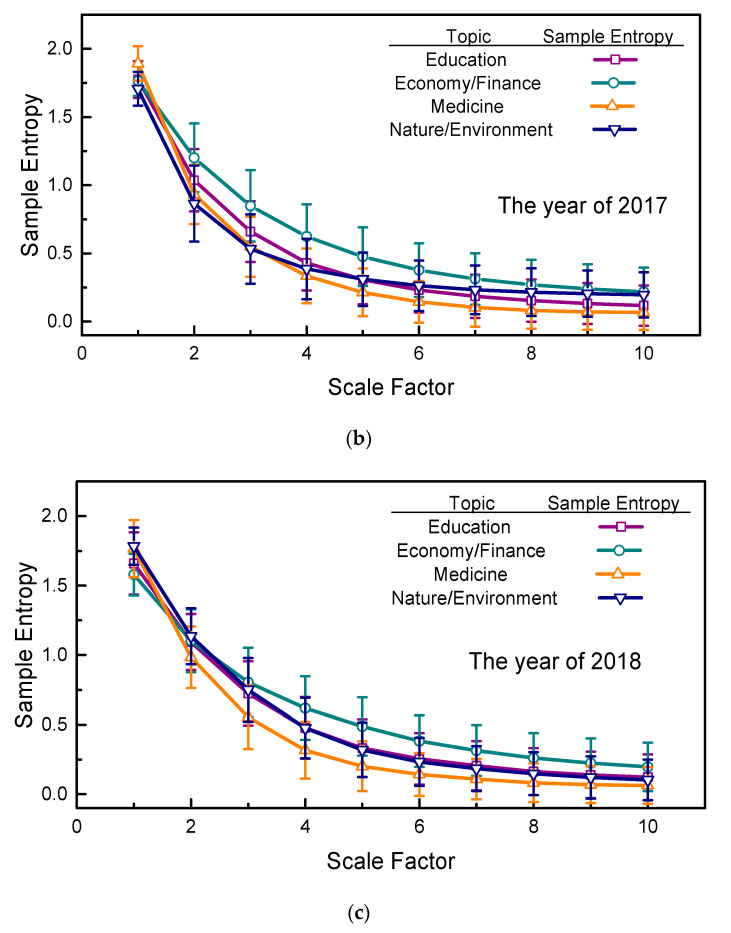
Comparison of sample entropies between the four selected topics: (**a**) 2016; (**b**) 2017; and (**c**) 2018.

We have focused on three years’ variations in sample entropies for selected topics. A horizontal comparison of the sample entropies with a 95% confidence interval of the four selected topics was also conducted, and the results of 2016, 2017, and 2018 are presented in Figure 5, in which it can readily be seen that the sample entropies of the four selected topics exhibit the same tendencies across years. That is, at large scale factors, the sample entropies of the four selected topics are quantitatively ranked as follows: economy/finance has the largest sample entropies, followed by education, nature/environment, and medicine. Notably, in 2016, the gaps between these quantitatively ranked curves are more obvious. These results suggest that human website searching activities on economy/finance topics are the most complicated and that elements in this page view series are less temporally correlated when compared to the other three topics.

Comparing the rate of descent of the curves in Figure 5, the sample entropies for the topic of medicine show the most rapid decrease as the scale factor increases, which shows that the elements of the page view series for medicine are the most strongly temporally correlated among the four selected topics. In Figure 3, note that no obvious outlier occurred in the page view series for medicine either, and the smoothness of these curves may accelerate their descent. Hence, this topic’s smallest sample entropies at larger scale factors and its most rapid descent suggests that the human website searching activities related to medicine are more strongly temporally correlated and more regular than those of the other three selected topics. Again, as in previous discussions of Figure 4, the sMSE algorithm highlights the results shown in Figure 5 at large scale factors, in which chaotic page view series are ordered and regularized.

It should be noted that, above, the estimated sample entropies (mean value) using the sMSE algorithm are compared. Once the 95% confidence interval is involved, the comparative results should be furtherly validated using statistical analysis. In what follows, we conduct an assessment of significance of difference for the comparative results of Figure 4 and Figure 5. For comparative results in Figure 4, for each selected topic, the Wilcoxon signed-rank test is selected to access the significance of difference for the sample entropies with a 95% confidence interval in different years. For Figure 5, for a given year, the Mann-Whitney *U*-test is applied to compare different selected topics. Without loss of generality, a p<0.05 is considered to be significant. Both the Wilcoxon signed-rank test and the Mann-Whitney *U*-test is realized by using Matlab R2018a.

For the selected topics, the *p* values of the Wilcoxon signed-rank test for Figure 4 with different year pairs are given in Table 2. It readily can be seen in Table 2 that, for education, the *p*-values of all year pairs are smaller than 0.05. The variations of sample entropy in Figure 4a are therefore considered to be significantly different, whereas for the other three topics, the *p* values are all larger than 0.05, except the sample entropies for economy/finance topics in the years of 2017 and 2018. Looking back to the sample entropies in Figure 4, although the variations of sample entropy of the economy/finance and the nature/environment topics are complicated, the statistical analysis results suggest the sample entropies of these two topics should be regarded as the same in the past three years. Specially, for medicine topics, the large *p* values in Table 2 show agreement with the invariant sample entropies in Figure 4c in the past three years. Therefore, by combining the results in Figure 4 and Table 2, the variations of sample entropy are acceptable and feasible for analyzing the complexity of the education and medicine topics, whereas for topics of economy/finance and natural/environment, the sample entropies in Figure 4 should be treated as undistinguished. Similarly to Table 2, the *p* value of the Mann-Whitney *U*-test for sample entropies in Figure 5 are presented in Table 3, Table 4 and Table 5, respectively. The *p* values in Table 3, Table 4 and Table 5 are all found to be larger than 0.05, which suggests that the sample entropies of different selected topics in Figure 5 are the same with regard to statistics. The complexity of different selected topics is therefore regarded to be undistinguished in Figure 5.

By taking the results of sample entropies and statistical analysis, the sample entropies, which are estimated by the sMSE algorithm, are found to be feasible for analyzing the temporal variations of complexity of page views of certain topics, for example, education and medicine. However, it can not be simply applied when analyzing the temporal variations of the complexity of page views over different topics.

Potential explanations are, firstly, that the complexities of the selected topics in past three years are undistinguished as is. Secondly, it should be noted that we sum up all the page view data in subcategories Level 2 as the total page views of the topic in subcategories Level 1, since the page view data in subcategories Level 2 may be independent of each other. Based on the law of large numbers, once summation is conducted, the total distribution of the four selected topics in subcategories Level 1 can be regarded as normal. Lastly, as we mentioned in Section 2, the selection of tolerance *r* affects the sample entropy estimates clearly, and probably leads to the undistinguished sample entropy estimates over the four selected topics. We therefore suggest these paths for forthcoming studies:

(1) On data processing, the way of collecting page view data should be considered carefully, subcategories in low levels in Figure 2 should probably be investigated separately;
(2) On methodology, with regard to the MSE algorithm, the difference between two ways of selecting the threshold value *r* should be investigated for more accurate and robust results;
(3) On background, for topics, which can be feasibly analyzed by sample entropy, the variations and explanations of complexity may be related to certain social issues, if possible.

## 5. Conclusions

This paper attempts to examine the complexities and temporal correlations of page views of four selected topics on Wikipedia using an sMSE algorithm. Sample entropies of the four selected topics are compared to reveal their temporal variations, showing vivid variations between different topics in three recent years. Meanwhile, the complexities of the page views of the selected topics are investigated and regular variations in the sample entropies of different topics are also found. Statistical analysis is then conducted to validate the variations, and the results suggest the sample entropy estimated by the sMSE is feasible in analyzing the temporal variations of the complexity of page view data for some topics. However, the regular variations of sample entropy can’t be simply accepted as is when different topics are compared. Potential explanations are given and discussed, and paths for forthcoming studies are also suggested.

## Figures and Tables

**Table 1:** Characteristics of the collected data.

Topics	Number of Subcategories	Year	Length	Mean Value
Medicine	28	2016	366	212,281
		2017	365	199,207
		2018	359	198,467
Education	27	2016	366	312,365
		2017	365	319,447
		2018	359	298,771
Economy/finance	32	2016	366	354,896
		2017	365	323,208
		2018	359	293,831
Nature/environment	16	2016	366	145,095
		2017	365	148,254
		2018	359	110,283

**Table 2:** *p* values of the Wilcoxon signed-rank test for results in Figure 4.

Year Pairs *p-Value* Topic	(2016,2017)	(2016,2018)	(2017,2018)
**Education**	0.0273	0.0488	0.0137
**Economy/finance**	0.4922	0.0840	0.0039
**Medicine**	0.4922	0.6250	1.0000
**Nature/environment**	0.8457	0.4316	0.9219

**Table 3:** *p* values of the Mann-Whitney *U*-test for results in Figure 5a.

Topic Paris	(Edu, E/F)	(Edu, Med)	(Edu, N/E)	(E/F, Med)	(E/F, N/E)	(Med, N/E)
***p*-value**	0.4727	0.1859	0.5205	0.0757	0.2730	0.3847

**Table 4:** *p* values of the Mann-Whitney *U*-test for results in Figure 5b.

Topic Paris	(Edu, E/F)	(Edu, Med)	(Edu, N/E)	(E/F, Med)	(E/F, N/E)	(Med, N/E)
***p*-value**	0.2123	0.3447	0.5708	0.1041	0.2703	0.2413

**Table 5:** *p* values of the Mann-Whitney *U*-test for results in Figure 5c.

Topic Paris	(Edu, E/F)	(Edu, Med)	(Edu, N/E)	(E/F, Med)	(E/F, N/E)	(Med, N/E)
***p*-value**	0.4274	0.2413	0.7913	0.1212	0.2447	0.3847
